# The Absence of Nrf2 Enhances NF-**κ**B-Dependent Inflammation following Scratch Injury in Mouse Primary Cultured Astrocytes

**DOI:** 10.1155/2012/217580

**Published:** 2012-02-22

**Authors:** Hao Pan, Handong Wang, Xiaoliang Wang, Lin Zhu, Lei Mao

**Affiliations:** Department of Neurosurgery, Jinling Hospital, School of Medicine, Nanjing University, Nanjing, Jiangsu 210002, China

## Abstract

It has been proved that Nrf2 depletion enhances inflammatory process through activation of NF-**κ**B in the brain after TBI, but little is known about the relationship between Nrf2 and NF-**κ**B in astrocytes after TBI. Hence, we used primary cultured astrocytes from either Nrf2 wildtype or knockout mice to study the influence of Nrf2 on the activation of NF-**κ**B and expression of proinflammatory cytokines in a model of TBI *in vitro*. Primary cultured astrocytes were scratched to mimic the traumatic injury *in vitro*. Then the DNA-binding activity of NF-**κ**B was evaluated by EMSA. The mRNA and protein levels of TNF-**α**, IL-1**β**, IL-6, and MMP9 were also evaluated. Gelatin zymography was performed to detect the activity of MMP9. The activity of NF-**κ**B and expression of proinflammatory cytokines mentioned above were upregulated at 24 h after scratch. The expression and activity of MMP9 were also elevated. And such tendency was much more prominent in Nrf2 KO astrocytes than that in WT astrocytes. These results suggest that the absence of Nrf2 may induce more aggressive inflammation through activation of NF-**κ**B and downstream proinflammatory cytokines in astrocytes.

## 1. Introduction

Brain damage following traumatic injury is a result of direct (primary injury) and indirect (secondary or delayed injury) mechanisms. The secondary injury mechanism involves the initiation of an acute inflammatory response, including breakdown of blood-brain barrier (BBB), brain edema, infiltration of peripheral blood cells, and activation of resident immunocompetent cells, as well as the release of numerous immune mediators such as interleukins and chemotactic factors [1]. And the subsequent inflammation leads to secondary damage not only in brain but also in other organs [2–4].

Nuclear factor erythroid 2-related factor2 (Nrf2) is a transcription factor that regulates many kinds of antioxidant genes. Several studies have demonstrated that Nrf2 regulates the inflammation in the brain after traumatic brain injury (TBI). It was also showed that TBI could induce more aggravated damage in Nrf2 knockout mice than in wildtype mice [5]. On the contrary, such inflammation can be extenuated through elevating the level of Nrf2 by its inducers, such as sulforaphane (SFN) [6]. It is well documented that astrocytes play a critical role in maintaining normal brain physiology and responding to injury or disease. In fact, all the aforementioned studies are performed *in vivo*. Little is known about the relationship between Nrf2 and inflammation in astrocytes after TBI. Therefore, here we analysed the influence of Nrf2 on the expression of proinflammatory cytokines in primary cultured astrocytes from transgenic mice after scratch injury.

## 2. Methods and Materials

### 2.1. Primary Culture and Identification of Mouse Astrocytes

Nrf2 knockout ICR mice were kindly provided by Dr. Thomas W. Kensler (Johns Hopkins University, Baltimore, MD, USA). Primary astrocytes were obtained from postnatal 2-day-old Nrf2 wide type (WT) and knockout (KO) mice (6 mice for each genotype). Following decapitation, the cortices were dissected out, and the meninges and associated blood vessels were removed. The tissue was roughly chopped with a scalpel blade, incubated in 0.5% trypsin for 10 minutes at 37°C, and agitated every few minutes. After digestion, the tissue was rinsed twice in DMEM with 10% FBS, followed by a mechanical dissociation in DMEM with 20% FBS and 5 units/mL penicillin, 5 *μ*g/mL streptomycin (complete culture medium). After incubation for 1 h, the supernatant was transferred to a new flask or dish (Costar, USA) for depleting the residual epithelial cells. Then the cells were cultivated at 37°C with 5% CO_2_. And the complete culture medium was half-changed twice a week. Astrocytes expanded for about 7 days to reach confluence. Then the flasks or dishes were shaken at 150 rpm for 4 h to deplete the microglia and less adherent cells from the cultures. After shaking, the resulting cultures were mainly astrocytes, which were determined by immunoreactivity for GFAP (sc-166481, Santa Cruz Biotechnology, CA, USA). Cells passaged for 2-3 generations were used in the following studies.

### 2.2. In Vitro Model of TBI Established by Scratch Injury

Astrocyte scratch injury was performed as in a previous report [7]. Astrocytes were planted in 6-well plates and grown to confluence. The cell monolayer was scratched with a sterile 26G syringe needle, resulting in the formation of a 0.5 mm wide gap. Immediately after scratch, cells were washed twice with sterile PBS, cultured with complete culture medium, and named as TBI group. Cells, which did not received scratch, were used as blank control and named as sham group.

### 2.3. Cell Death Measurement by CCK-8 Assay

1 × 10^3^/well cells were seeded to 96-well culture plates and cultivated for 24 h to adhere. Then the cell was scrated as mentioned above. Astrocyte cell death was assessed by cell counting kit-8 (CCK-8) (Dojindo, Japan) assay 24 h after scratch according to the manufacturer's protocol. Briefly, 10 *μ*L CCK-8 was added into every well and incubated for 1 h. Then OD value was read at 450 nm using a Bio-Rad ELISA microplate reader (Bio-Rad Laboratories, CA, USA). All measurements were performed in sextuplicate. Results were expressed as mean of OD value at 450 nm ± SD.

### 2.4. Electrophoretic Mobility Shift Assay (EMSA)

Monolayers of astrocytes were washed with PBS and harvested by scraping into cold PBS. The cell pellet obtained by centrifugation was resuspended in buffer containing 10 mM HEPES (pH 7.9), 10 mM KCl, 0.1 mM EDTA, 0.1 mM EGTA, 1.0 mM DTT, and 0.5 mM phenylmethylsulfonyl fluoride. Then 10% Nonidet P-40 was added and vortexed briefly, and the nuclei were pelleted by centrifugation. The nuclear proteins were extracted with buffer containing 20 mM HEPES (pH 7.9), 0.4 mM NaCl, 1.0 mM EDTA, 1.0 mM EGTA, 1.0 mM DTT, and 1.0 mM phenylmethylsulfonyl fluoride. Insoluble material was removed by centrifugation at 14000 rpm, and the supernatant containing the nuclear proteins was stored at –80°C until use. Protein concentration was determined using a bicinchoninic acid assay kit with bovine serum albumin as the standard (Pierce Biochemicals, Rockford, IL, USA). EMSA was performed using gel shift assay system (Promega, Madison, WI, USA). Consensus oligonucleotide probe (5′-AGTTGAGGGGACTTTCCCAGGG-3′) was end-labeled by T4-polynucleotide kinase using [*γ*-32P]-ATP (Free Biotech., Beijing, China). Nuclear protein (20 *μ*g) was preincubated in 20 *μ*L binding buffer containing 10 mM Tris-HCl (PH 7.5), 1 mM MgCl_2_, 0.5 mM NaCl, 4% glycerol, 0.5 mM EDTA, 0.5 mM DTT, and 2 *μ*g poly dI-dC for 20 minutes on ice. After addition of the 1 *μ*L 32P-labled oligonucleotide probe, the incubation was continued for 20 minutes on ice. The DNA-protein complexes were separated by electrophoresis on 4% nondenaturing polyacrylamide gel in 0.5 × TBE buffer (*tris*-borate-EDTA) at 390 V for 1 hour at 4°C. After electrophoresis, the gel was dried and exposed to X-ray film (Fuji Hyperfilm, Tokyo, Japan). Levels of NF-*κ*B DNA binding activity were quantified by software ImageJ. 

### 2.5. RT-PCR

Total RNA was isolated with Trizol (Invitrogen, CA, USA), and single-stranded cDNA was synthesized from 2 *μ*g of total RNA with BU-Script RT-Kit (Biunique, Jiangsu, China) according to the manufacturer's protocol. The cDNA was stored in −20°C. Reverse transcription was conducted with GoTaq Green Master Mix (Promega, WI, USA) according to the manufacturer's protocol.  Table 1 shows the primers and PCR parameters. PCR products were detected by agarose gel electrophoresis. The intensity of the bands was analyzed by ImageJ program. The level of *β*-actin was used as an internal standard.

### 2.6. Western Blot

To obtain total protein lysates, cells were homogenized in RIPA buffer (1% NP40, 0.5% sodium deoxycholate, 0.1% SDS, 1 mM EDTA, 1 mM EGTA, 1 mM Na_3_VO_4_, 20 mM NaF, 0.5 mM DTT, 1 mM PMSF, and protease inhibitor cocktail in PBS pH 7.4) and centrifuged at 12,000 g for 15 min at 4°C. Protein concentrations were estimated by Coomassie Plus Protein Assay Reagent (Pierce, IL, USA). Fifty micrograms of the resulting cytosolic protein extracts were heat-denatured in Laemmli sample loading buffer, separated by 10% sodium dodecyl sulfate polyacrylamide gel electrophoresis, and electroblotted onto a nitrocellulose membrane. For immunoblotting, membranes were blocked with 5% nonfat dry milk in saline buffer overnight at 4°C, and the following antibodies were used: anti-*β*-actin (sc-130657, Santa Cruz Biotechnology, CA, USA, 43 kDa) and anti-matrix metallopeptidase 9 (MMP9) (sc-6841, Santa Cruz Biotechnology, CA, USA, 92 kDa). Each primary antibody was diluted appropriately in blocking buffer and then added to the blots for 1 h at room temperature. The blots were washed three times in the washing buffer and covered with the horseradish peroxidase-linked secondary antibody at a 1 : 2000 dilution for 1 h. Blots were incubated with enhanced chemiluminescence (ECL) detection system (Amersham Biosciences, Bucks, UK) and exposed to radiographic film (Fuji Hyperfilm, Tokyo, Japan). ImageJ was used to analyze the intensity of the blots. The level of *β*-actin was used as internal standard.

### 2.7. Enzyme-Linked Immunosorbent Assay (ELISA)

Cells of four groups were homogenized as mentioned above. The supernatant was collected, and total protein was determined by Coomassie Plus Protein Assay Reagent (Pierce, IL, USA). Levels of tumor necrosis factor-alpha (TNF-*α*), interleukin-1 beta (IL-1*β*), and interleukin-6 (IL-6) protein were quantified using ELISA kits specific for mouse according to the manufacturer's instructions (Bender MedSystems Inc. CA, USA). Briefly, prepared the standard and created the standard dilution for building standard curve. Then samples and biotinconjugate were added to microwell strips. After incubated for 2 h at room temperature, the microwell strips were washed 3 times with wash buffer, and streptavidin-HRP were added to all wells. After incubated for 1 h, microwell strips were washed 3 times followed by adding TMB substrate. After incubated for about 10–30 min, the stop solution was added. The colour intensity was measured at 450 nm using a Bio-Rad ELISA microplate reader (Bio-Rad Laboratories, CA, USA). The concentration of protein was determined according to the standard curve and expressed as pg/mg of total protein. 

### 2.8. Gelatine Zymography

Cells of four groups were homogenized in lysis buffer containing 50 mM Tris-HCl (pH 7.4), 150 mM NaCl, 5 mM CaCl_2_, 0.2 mM NaN_3_, and 0.01% Triton. Soluble extracts were separated by centrifugation and stored at –20°C. Gelatin zymography was performed according to the manufacturer's instructions (Genmed Scientifics Inc, MA, USA). Briefly, 40 *μ*g cytosolic protein extracts were separated by electrophoresis. Then the proteins were renatured by incubation in 2.5% Triton X-100 and then incubated in substrate buffer for 40 h at 37°C to enable the MMP9 to cleave the gelatin. After rinsing in water, each gel was stained with Coomassie blue for 1 h and destained in 50% methanol. Proteolytic activities were showed by clear bands in blue gel which indicates the lysis of the substrate. Quantification of MMP9 band density was performed with image analysis program ImageJ. 

### 2.9. Statistical Analysis

Data were expressed as mean ± SD and evaluated by ANOVA and LSD multiple comparison test. *P* values <0.05 were considered to be significant. All analyses were performed by using SPSS 18.0 software. 

## 3. Results

### 3.1. Depletion of Nrf2 Aggravated the Cell Death in Astrocytes after Scratch Injury

**Figure 1:**
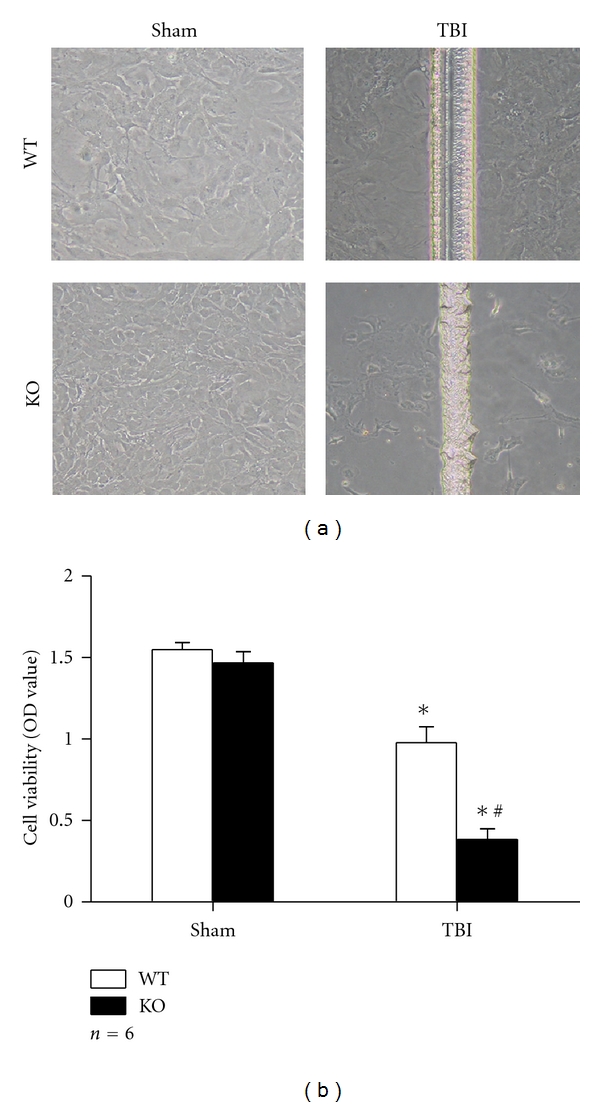
Loss of Nrf2 enhanced cell death after scratch. (a) Cultured astrocytes scratched or not were observed under microscope (×100). There was more cell death in group KO TBI. (b) CCK-8 analysis revealed lower OD value in scratched astrocytes (*P* < 0.01). Group KO TBI showed significant lower OD value than group WT TBI (*P* < 0.01). (*P < 0.01, compared with sham counterpart; ^#^ *P* < 0.01, compared with WT TBI).

Cell death was detected by microscope and CCK-8 analysis at 24 h after scratch. The detachment from culture plate, cell lost, and cytoplasmic process distortion were mostly observed in both sides of scratch line in group KO TBI. CCK-8 assay also showed lower OD value of group KO TBI (0.38 ± 0.064) than that of group WT TBI (0.98 ± 0.098) (*P *< 0.01), which also suggested more cell death in group KO TBI (Figure 1). There was no difference between two sham-operated groups.

### 3.2. Disruption of Nrf2 Enhanced Upregulation of NF-*κ*B DNA-Binding Activity after Scratch Injury

**Figure 2:**
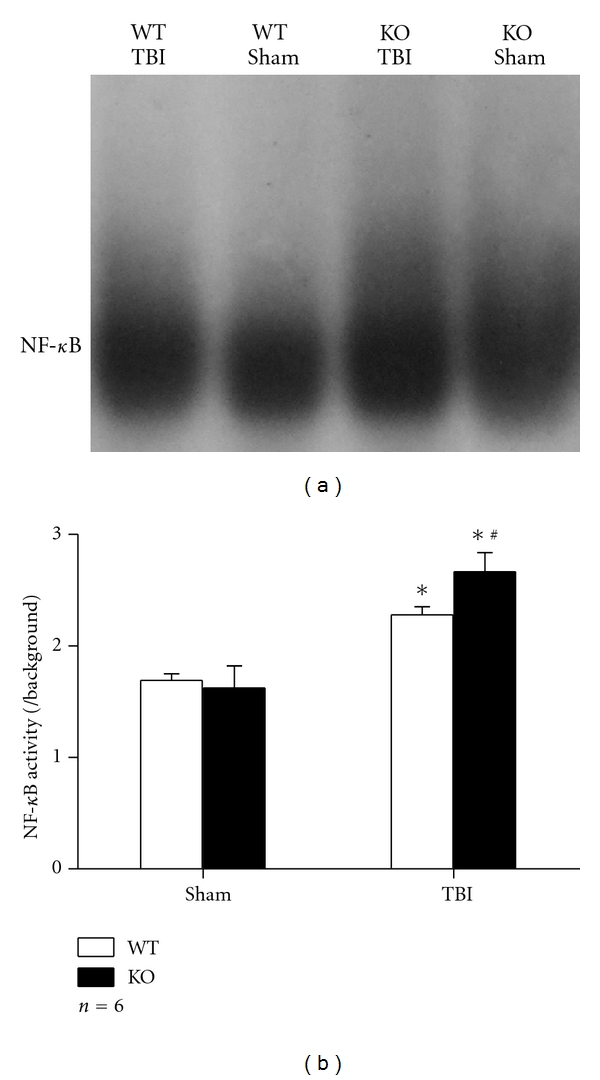
EMSA analysis for DNA-binding activity of NF-*κ*B. (a) Representative film shows DNA-binding activity of NF-*κ*B in Nrf2 WT or KO astrocytes 24 h after scratch or not. (b) Quantification of NF-*κ*B DNA-binding activity was performed by ImageJ. NF-*κ*B DNA-binding activity in astrocytes increased after scratch (*P* < 0.01) and was prominently greater in group KO TBI than in group WT TBI (*P* < 0.01). (*P < 0.01, compared with sham counterpart; ^#^ *P* < 0.01, compared with WT TBI).

It has been reported that NF-*κ*B is activated in brain after TBI [2]. Here, we studied the DNA-binding activity of NF-*κ*B of astrocytes from Nrf2 WT or KO mice by EMSA at 24 h after scratch injury. Scratch injury induced activation of NF-*κ*B in astrocytes of both genotypes, while higher NF-*κ*B activity was observed in group KO TBI than in group WT TBI (2.67 ± 0.173 versus 2.28 ± 0.072, *P* < 0.01) (Figure 2).

### 3.3. Expression of Proinflammatory Cytokines Was Elevated in Nrf2 Knockout Astrocytes after Scratch Injury

**Figure 3:**
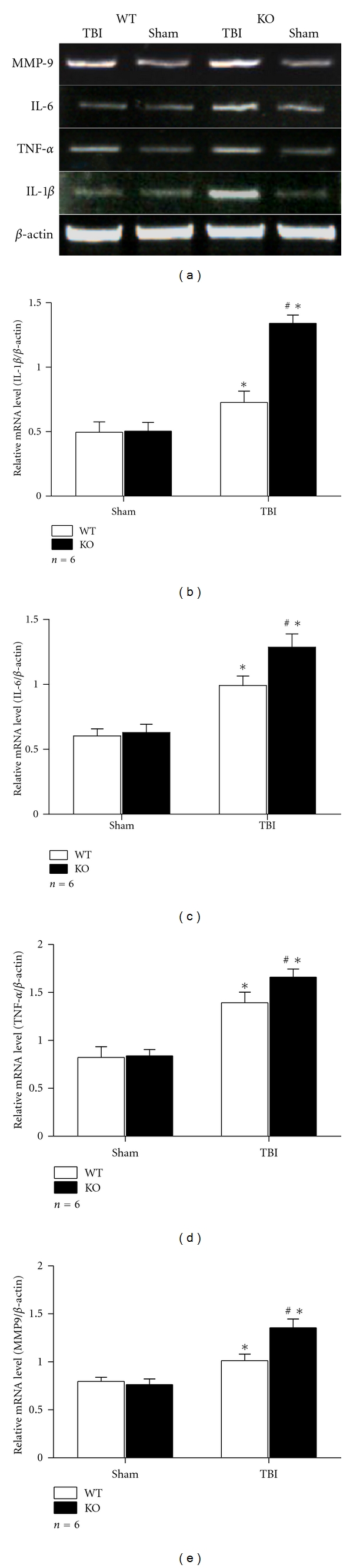
RT-PCR analysis of mRNA level of proinflammatory cytokines and MMP9. (a) RT-PCR showed that mRNA level of TNF-*α*, IL-1*β*, IL-6, and MMP9 was elevated in astrocytes after scratch and was significantly higher in group KO TBI than in group WT TBI. (b–e) Quantitative analysis of RT-PCR results showed that relative mRNA level of proinflammatory cytokines mentioned above and MMP9 was higher in astrocytes after scratch (*P* < 0.01). Such elevation was more severe in group KO TBI than in group WT TBI (*P* < 0.01).  (*P < 0.01, compared with sham counterpart; ^#^ *P* < 0.01, compared with WT TBI).

**Figure 4:**
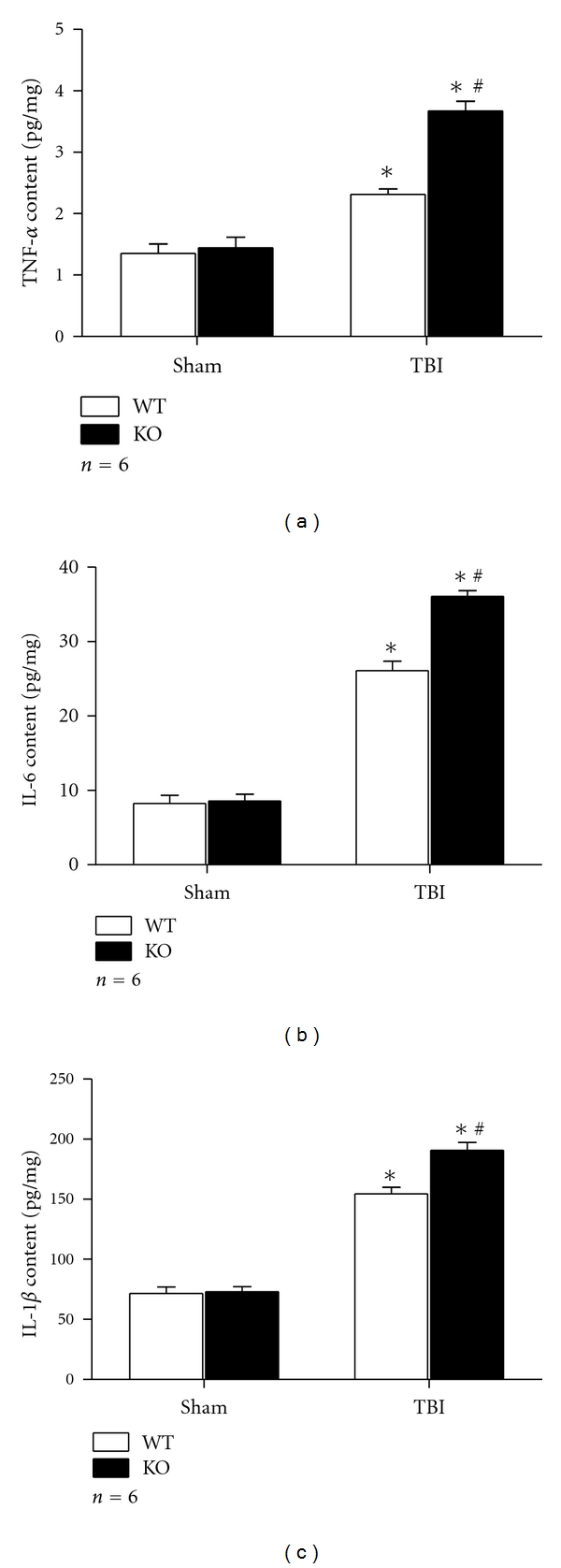
ELISA analysis of protein level of proinflammatory cytokines. ELISA results revealed protein level of these cytokines were elevated in astrocytes after scratch as compared with sham control (*P* < 0.01). And they were much higher in Nrf2 KO astrocytes than in their WT counterparts (*P* < 0.01). (a) TNF-*α*. (b) IL-6. (c) IL-1*β*  (*P < 0.01, compared with sham counterpart; ^#^ *P* < 0.01, compared with WT TBI).

As we all know, TNF-*α*, IL-1*β*, and IL-6 are regulated by NF-*κ*B and reflect the endogenous activity of NF-*κ*B. It has been revealed that these proinflammatory cytokines are increased after TBI in animals [2, 8]. And our previous study has proved that higher levels of such proinflammatory cytokines were observed in the brain of Nrf2-deficient mice after TBI [5]. But it is still ambiguous about the relationship between these cytokines and Nrf2 in astrocytes after TBI. Here, we tested the levels of TNF-*α*, IL-1*β*, and IL-6 in cultured astrocytes at 24 h after scratch injury by RT-PCR and ELISA. It was shown that the mRNA levels of TNF-*α*, IL-1*β*, and IL-6 were increased after scratch in both WT and KO astrocytes when compared with their sham counterparts, respectively. Moreover, the mRNA levels of these cytokines in group KO TBI were much higher than those in group WT TBI (1.66 ± 0.085 versus 1.39 ± 0.110 for TNF-*α*, 1.34 ± 0.064 versus 0.73 ± 0.088 for IL-1*β*, and 1.28 ± 0.102 versus 0.99 ± 0.073 for IL-6. *P* < 0.01) (Figures 3(a)–3(d)). The results of ELISA revealed that the protein level of TNF-*α* was upregulated after scratch injury in WT and KO astrocytes when compared with their sham counterparts. And it was also higher in group KO TBI than in group WT TBI (3.67 ± 0.156 versus 2.31 ± 0.087, *P* < 0.01) (Figure 4(a)). Similar tendency was observed in protein level of IL-6 and IL-1*β*. For IL-6, it was 36.07 ± 0.786 for group KO TBI while 25.76 ± 0.536 for group WT TBI (*P* < 0.01) (Figure 4(b)). For IL-1*β*, it was 190.75 ± 6.339 for group KO TBI while 154.50 ± 5.348 for group WT TBI (*P* < 0.01) (Figure 4(c)).

### 3.4. Expression and Activity of MMP9 Were Greatly Enhanced in Nrf2 Knockout Astrocytes after Scratch Injury

**Figure 5:**
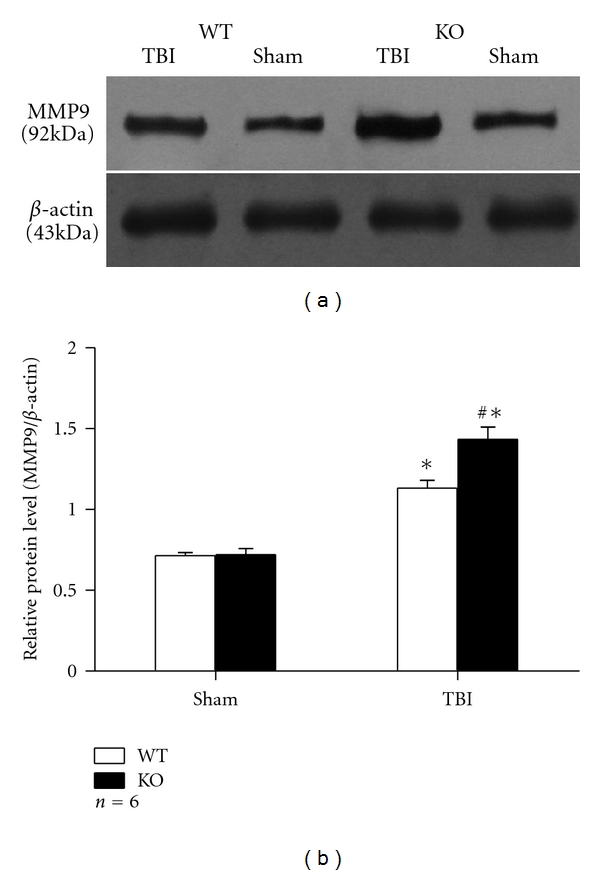
Western blot analysis of MMP9 protein level. (a) Western blot showed that protein level of MMP9 was elevated in astrocytes after scratch and was significantly higher in group KO TBI than in group WT TBI. (b) Quantitative analysis of western blot result showed that relative protein level of MMP9 was higher in astrocytes after scratch (*P* < 0.01). Such elevation was much severe in group KO TBI than in group WT TBI (*P* < 0.01). (*P < 0.01, compared with sham counterpart; ^#^ *P* < 0.01, compared with WT TBI).

**Figure 6:**
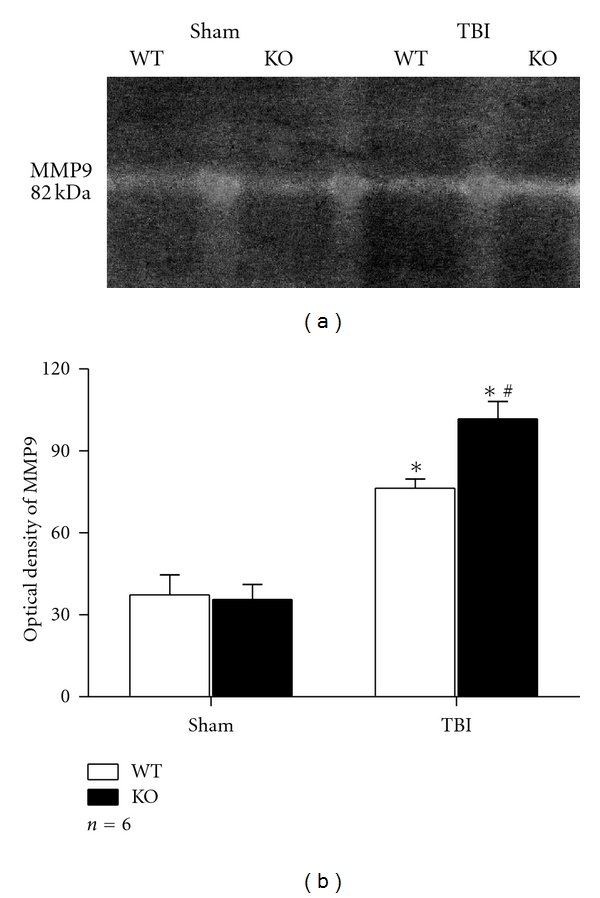
Gelatin zymography of MMP9 activity. (a) Gelatin zymography analysis showed higher MMP9 activity in group KO TBI than in group WT TBI. (b) Quantitative analysis showed that MMP9 activity was higher in astrocytes after scratch (*P* < 0.01). Such elevation was much severe in group KO TBI than in group WT TBI (*P* < 0.01). (*P < 0.01, compared with sham counterpart; ^#^ *P* < 0.01, compared with WT TBI).

MMP9 is an important gelatinase to induce or aggravate the inflammation process. In the present study, we elevated the mRNA, protein levels, and activity of MMP9 in astrocytes at 24 h after scratch injury by RT-PCR, western blot, and gelatine zymography. Expression of MMP9 was elevated after scratch injury in WT and KO astrocytes as compared with their sham counterparts. The mRNA level of MMP9 was higher in group KO TBI than in group WT TBI (1.36 ± 0.090 versus 1.01 ± 0.068, *P* < 0.01) (Figures 3(a) and 3(e)). Significant difference was also discovered in MMP9 protein level by western blot, as 1.44 ± 0.076 for group KO TBI and 1.13 ± 0.048 for group WT TBI (*P* < 0.01) (Figure 5). Gelatine zymography revealed higher MMP9 activity in group KO TBI than that in group WT TBI too (101.76 ± 6.343 versus 76.32 ± 3.388, *P* < 0.01) (Figure 6). 

## 4. Discussion

The present study demonstrated that scratch injury induced the upregulation of NF-*κ*B DNA binding activity and overexpression of TNF-*α*, IL-1*β*, IL-6, and MMP9 in cultured astrocytes. Also we revealed that scratch injury induced higher activity of NF-*κ*B and enhanced expression of proinflammatory cytokines in Nrf2 knockout cultured astrocytes than those in wildtype astrocytes for the first time.

It has been demonstrated that NF-*κ*B is activated in brain and spinal cord after traumatic injury [2, 9]. As a transcript factor, NF-*κ*B binds with DNA once it was activated and induces transcription of MMP9 and a battery of proinflammatory cytokines, including TNF-*α*, IL-1*β*, and IL-6, in brain tissues after TBI [3, 10, 11]. Our results also revealed that scratch injury induced elevation of NF-*κ*B DNA binding activity, overexpression of MMP9, and proinflammatory mediators mentioned above in cultured astrocytes. It has been demonstrated that there is an autoregulatory loop among these proinflammatory mediators. For example, TNF-*α* and IL-1*β* are potent stimulators for MMP9 in astrocytes [12, 13], and IL-6 induces overexpression of MMP9 in human colon carcinoma cells [14], and TNF-*α* and IL-1*β* also can induce activation of NF-*κ*B [15, 16]. This autoregulatory loop extremely aggravates the damaging effect of inflammation and induces secondary injury to brain. It is a good choice for targeting on an upstream factor to prevent such autoregulatory loop after TBI. 

Nrf2-ARE pathway has been proved to be the key regulator in reducing oxidative stress, inflammatory damage, and accumulation of toxic metabolites, which are all involved in TBI. Our previous study has proved the augmentation of Nrf2 in brain tissue after TBI [17]. Enhanced level of Nrf2 activates transcription of a group of antioxidant genes, such as heme oxygenase-1 (HO-1) and NAD (P)H: quinone oxidoreductase-1 (NQO1), which would subsequently reduce the damage in brain [18]. Postinjury administration of SFN, an inducer of Nrf2, significantly improves spatial memory of rat and decreases the immunoreactivity for 4-Hydroxynonenal (4-HNE), a marker of lipid peroxidation, in the cortex and the CA3 subfield of hippocampus after TBI [19]. On the other hand, Nrf2-deficient mice appear more susceptible to TBI. Depletion of Nrf2 induces higher expression of proinflammatory mediators in brain after TBI [20]. Here, our results revealed that overexpression of TNF-*α*, IL-1*β*, IL-6, and MMP9 after scratch injury was more aggravated in cultured Nrf2 knockout astrocytes than in wildtype astrocyetes for the first time, and overexpression of these proinflammatory mediators led to more astrocytes deaths.

Data obtained from animal studies suggest the possibility that antioxidant effect of Nrf2 may be achieved by suppression of proinflammatory pathways which are mediated by NF-*κ*B signaling. Administration of SFN is found to be able to inhibit IKK/I*κ*B phosphorylation and p65 NF-*κ*B subunit nuclear translocation, consequently alleviating NF-*κ*B signaling [21]. And NF-*κ*B activation induced by lipopolysaccharide (LPS) could be attenuated by diverse Nrf2 activators, such as SFN and curcumin (CUR) [22]. Furthermore, our previous studies indicate that depletion of Nrf2 induces augmentation of NF-*κ*B activity and inflammatory response in lung, brain, and intestine after TBI [4, 5, 23]. Results from this study further confirmed such relationship existed in cultured astrocytes after scratch injury. Enhanced activation of NF-*κ*B is also discovered in lung, macrophages, and mouse embryonic fibroblasts of Nrf2-deficient mice after experimental sepsis [24]. Interestingly, it is reported that NF-*κ*B can inhibit Nrf2 at transcriptional level. NF-*κ*B p65 subunit repressed the Nrf2-ARE pathway at transcriptional level by competitive interaction with the CH1-KIX domain of CBP or local histone hypoacetylation [25]. All these findings indicate the potential complicate crosstalk between NF-*κ*B and Nrf2, which may be regulated by the upstream mitogen-activated protein kinase (MAPKs) pathway [26].

It has been confirmed that Nrf2 is mainly detected in nucleus of astrocytes after TBI [18]. In view of the fact that Nrf2 is a transcription factor which should take function mainly in nucleus, it can be reasoned that astrocytes may be one kind of respondent cells in activation of Nrf2-ARE pathway after TBI. Previous study identified the expression of TNF-*α*, IL-1*β*, and IL-6 in cultured astrocytes after treatment with LPS or oxyhemoglobin [27, 28]. Another study revealed that after middle cerebral artery occlusion, MMP9-positive astrocytes were observed in brain tissues by immunohistochemistry [29]. Those results indicate the role of astrocytes in inflammatory process after brain injury. But till now, there is no study focused on the relationship among astrocytes, Nrf2, and proinflammatory mediators after TBI. Our results demonstrated the upregulated expression of TNF-*α*, IL-1*β*, IL-6, and MMP9 in Nrf2 knockout astrocytes after scratch injury for the first time.

In conclusion, depletion of Nrf2 induced the activation of NF-*κ*B and the expression of TNF-*α*, IL-1*β*, IL-6, and MMP9 resulting in more cell deaths in astrocytes after scratch injury. These results suggest that Nrf2 may be an important target for anti-inflammatory therapy after TBI.

## Figures and Tables

**Table 1:** Primers and parameters used in RT-PCR.

Name	Primer		Tm (°C)	Cycle
	Forward	Reverse		
MMP9	5′-CTACTCTGAAGACTTGCCG-3′	5′-CCATACAGTTTATCCTGGTC-3′	57	35
TNF-*α*	5′-ACGGCATGGATCTCAAAGAC-3′	5′-GGTCACTGTCCCAGCATCTT-3′	55	30
IL-1*β*	5′-GAGTGTGGATCCCAAGCAAT-3′	5′-CTCAGTGCGGGCTATGACCA-3′	53	32
IL-6	5′-AGTTGCCTTCTTGGGACTGA-3′	5′-GCCACTCCTTCTGTGACTCC-3′	55	32
*β*-actin	5′-AGTGTGACGTTGACATCCGTA-3′	5′-GCCAGAGCAGTAATCTCCTTCT-3′	55	35
